# The AP-3 adaptor complex mediates sorting of yeast and mammalian PQ-loop-family basic amino acid transporters to the vacuolar/lysosomal membrane

**DOI:** 10.1038/srep16665

**Published:** 2015-11-18

**Authors:** Elisa Llinares, Abdoulaye Oury Barry, Bruno André

**Affiliations:** 1 Molecular Physiology of the Cell, Université libre de Bruxelles (ULB), IBMM, Gosselies, Belgium

## Abstract

The limiting membrane of lysosomes in animal cells and that of the vacuole in yeast include a wide variety of transporters, but little is known about how these proteins reach their destination membrane. The mammalian PQLC2 protein catalyzes efflux of basic amino acids from the lysosome, and the similar Ypq1, −2, and −3 proteins of yeast perform an equivalent function at the vacuole. We here show that the Ypq proteins are delivered to the vacuolar membrane via the alkaline phosphatase (ALP) trafficking pathway, which requires the AP-3 adaptor complex. When traffic via this pathway is deficient, the Ypq proteins pass through endosomes from where Ypq1 and Ypq2 properly reach the vacuolar membrane whereas Ypq3 is missorted to the vacuolar lumen via the multivesicular body pathway. When produced in yeast, PQLC2 also reaches the vacuolar membrane via the ALP pathway, but tends to sort to the vacuolar lumen if AP-3 is defective. Finally, in HeLa cells, inhibiting the synthesis of an AP-3 subunit also impairs sorting of PQLC2 to lysosomes. Our results suggest the existence of a conserved AP-3-dependent trafficking pathway for proper delivery of basic amino acid exporters to the yeast vacuole and to lysosomes of human cells.

Cystinosis is a genetic disease with an estimated incidence of 1 case per 100,000–200,000 births. This disorder is characterized by intracellular accumulation of cystine, the disulfide of the amino acid cysteine^1^, in all tissues. Cystinosis is caused by mutations in the CTNS gene encoding cystinosin, a lysosomal transporter catalyzing the export of cystine present in lysosomes^2^,^3^ (Fig. 1A). The only available treatment for cystinosis is oral doses and eyedrops of cysteamine, an aminothiol drug promoting long-term depletion of lysosomal cystine^4^. The mechanism of cystine depletion by cysteamine relies on entry of the drug into the lysosomes, followed by its reaction with cystine to form cysteine and the mixed disulfide cysteamine-cysteine. The latter compound, structurally similar to lysine, exits the lysosome via a basic amino-acid transporter named PQLC2, and once in the cytosol, it is reduced to cysteamine and cysteine by the glutathione system^5^,^6^ (Fig. 1B). Remarkably, PQLC2 belongs to the same transporter family as cystinosin. This family of seven-transmembrane-domain (7-TM) proteins is named “PQ-loop family”, because its members possess a duplicated region including a well-conserved PQ-dipeptide (Fig. 1C). This family belongs to a superfamily of transporters (the MtN3 clan in the Pfam database) which includes the 7-TM SWEET family sugar export proteins (including MtN3)^7^ and also 3-TM proteins such as the bacterial semiSWEETs and the mitochondrial pyruvate (MCP) transporters^8^,^9^. Recently, the first crystal structure of a bacterial semiSWEET transporter has been reported, revealing that the structural unit of these transporters is a triple helix bundle (THB)^10^. In 7-TM transporters such as cystinosin, PQLC2, and SWEET proteins, a linker TM (TM4) connects two THBs (Fig. 1C), whereas the 3-TM semiSWEET monomers associate into 6-TM dimers where the interface between the monomers provides the transport conduit^8^,^10^.

In the yeast *Saccharomyces cerevisiae*, the PQ-loop family comprises six members^11^. Among them, Ers1 is the closest yeast homologue of human cystinosin, and the phenotype of *ers1* mutants is reported to be complemented by cystinosin^12^. The Ypq1, −2, and −3 proteins, three other yeast PQ-loop transporters, are more similar in sequence to PQLC2 and localize to the membrane of the vacuole, the lysosome of yeast^6^. The *ypq2*∆ mutant is resistant to the toxic arginine analogue canavanine, a phenotype compatible with a role of Ypq2 in arginine (and canavanine) export from the vacuole. This phenotype is complemented by rat PQLC2, shown in electrophysiology and uptake experiments to catalyze export of basic amino acids and canavanine. This suggests that yeast Ypq2 and PQLC2 are functional orthologs^6^.

Previous work has shown that targeting of PQLC2 to the lysosome requires an acidic dileucine (285-EREPLL-291) present in its cytosolic C-terminal tail^6^. In the present study, we have used yeast as a model system to investigate the trafficking pathway by which PQ-loop proteins reach the vacuolar/lysosomal membrane. Our results show that the Ypq proteins preferentially use the pathway depending on the AP-3 adaptor complex and on an acidic dileucine motif present in their second cytosolic loop. When produced in yeast, the PQLC2 protein also reaches the vacuolar membrane via this AP-3-dependent pathway. The results of further experiments in HeLa cells reveal that the AP-3 complex is also essential to proper localization of PQLC2 to the lysosomal membrane.

## Results

### The Ypq1 protein can reach the vacuolar membrane via the ALP or endosomal pathway

**Figure 2:**
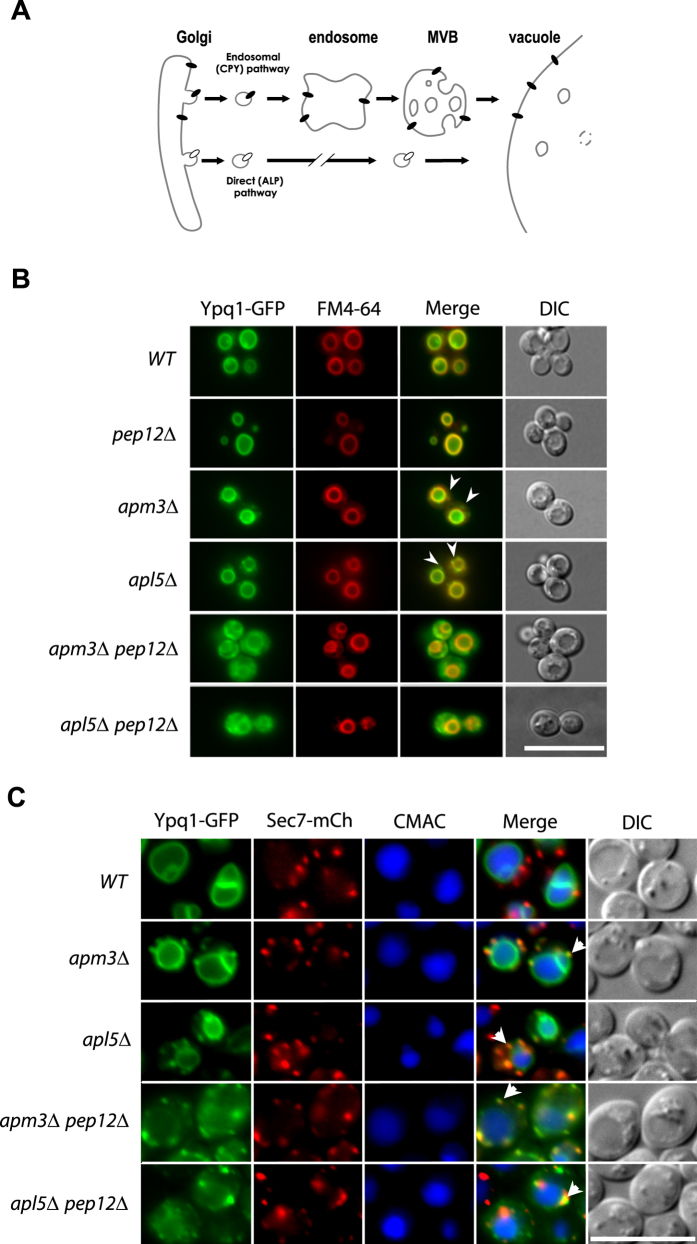
Ypq1 can reach the vacuolar membrane via the ALP or CPY pathways. (**A**) The two main trafficking pathways from the *trans*-Golgi to the vacuole in the yeast *Saccharomyces cerevisiae*. MVB: multivesicular body (**B**) Strains 23344c (*ura3*), EN046 (*pep12∆ ura3*), LL057 (*apm3∆ ura3*), LL061 (*apl5∆ ura3*), LL088 (*apm3∆ pep12∆ ura3*) and LL041 (*apl5∆ pep12∆ ura3*) transformed with the pLL063 (*YPQ1-GFP URA3*) plasmid were grown on a glucose-ammonium medium. Cells were allowed to internalize FM4-64 for 15 min to label the vacuole before imaging. (**C**) Strains GC004 (*ura3 leu2*), BOA003 (*apm3∆ ura3 leu2*), BOA005 (*apl5∆ ura3 leu2*), BOA007 (*apm3∆ pep12∆ ura3 leu2*), BOA008 (*apl5∆ pep12∆ ura3*) transformed with the pLL063 (*YPQ1-GFP URA3*) or pBOA010 (*SEC7-mCherry LEU2)* plasmids were grown on a glucose-ammonium medium. Cells were allowed to internalize CMAC for 30 min to label the vacuolar lumen before imaging. Scale bar: 10 μm. A quantification of the sublocalization patterns of Ypq1-GFP (**B**) and Ypq1-GFP-Sec7-mCherry (**C**) can be found as Supplementary Figure S1 online.

Having recently reported that a Ypq1-GFP fusion protein localizes to the vacuolar membrane^6^, we sought to determine by which trafficking route Ypq1 reaches this compartment. Vacuolar membrane proteins can reach the vacuole via two different pathways: the ALP (alkaline phosphatase) and the CPY (carboxypeptidase Y) pathway, the latter involving passage of the proteins via endosomes (Fig. 2A). To test whether Ypq1 follows the CPY pathway, we examined its intracellular distribution in a *pep12*Δ mutant lacking a t-SNARE involved in vesicle fusion with the late endosome^13^. In this mutant, Ypq1-GFP was found exclusively at the vacuolar membrane (Fig. 2B). In a *vps27*Δ mutant lacking a component of the endosomal ESCRT-0 complex, membrane proteins trafficking via endosomes are typically stacked in clearly visible class E compartments corresponding to abnormally enlarged endosomes, but the Ypq1-GFP protein was targeted normally to the vacuolar membrane in this mutant (data not shown). These results show that Ypq1 does not require a functional CPY pathway to reach the vacuole. We then tested whether Ypq1 reaches the vacuolar membrane via the ALP pathway. It is well documented that this pathway requires the AP-3 adaptor complex, assumed to act at the trans Golgi to sort cargo proteins into vesicles that subsequently fuse with the endosomes. This complex is a heterotetramer consisting of two large subunits (β3a and δ), a medium subunit (μ3a), and a small subunit (σ3), and the absence of any of these subunits results in a deficient AP-3 complex^14^. We therefore isolated two AP-3-deficient strains, *apm3*Δ (lacking subunit μ3a) and *apl5*Δ (lacking subunit δ). In these mutants, Ypq1-GFP was found at the vacuolar membrane as well as in small punctate structures readily labeled with FM4-64 and not observed in wild-type cells (Fig. 2B). In *amp3*Δ and *apl5*Δ mutant cells also expressing a functional Sec7-mCherry to label the Golgi, a high percentage of these punctate structures were decorated with Sec7-mCherry (Fig. 2C) (for a quantification of the sublocalization patterns, see Supplementary Fig. S1 online). These results indicate that when the ALP pathway is deficient, Ypq1 tends to accumulate in the Golgi while a significant fraction of the protein reaches the vacuolar membrane via another pathway. To test whether the latter is the CPY pathway, we determined the location of Ypq1-GFP in *apm3*Δ *pep12*Δ and *apl5*Δ *pep12*Δ double mutants (Fig. 2B). Interestingly, delivery of Ypq1-GFP to the vacuole was largely impaired in these strains, and the protein was found mostly dispersed throughout the cytosol as well as in punctate structures labeled with the Sec7-mCherry (Fig. 2B,C) (for a quantification of the sublocalization patterns, see Supplementary Fig. S1 online). The vacuole in these double mutants could be normally labeled with FM4-64. These results show that Ypq1 can be sorted to the vacuolar membrane via both the ALP and the CPY pathways. In wild-type cells, it uses mainly the ALP pathway, but when this pathway is deficient, the protein resides longer in the Golgi but can still efficiently reach the vacuolar membrane via the CPY pathway. When both the ALP and the CPY pathways are deficient, a small fraction of Ypq1-GFP is detectable at the vacuolar membrane, suggesting that the protein can use yet another pathway, but one that is much less efficient at properly targeting Ypq1 to the vacuole.

### An acidic dileucine motif promotes Ypq1 sorting to the ALP pathway

**Figure 3:**
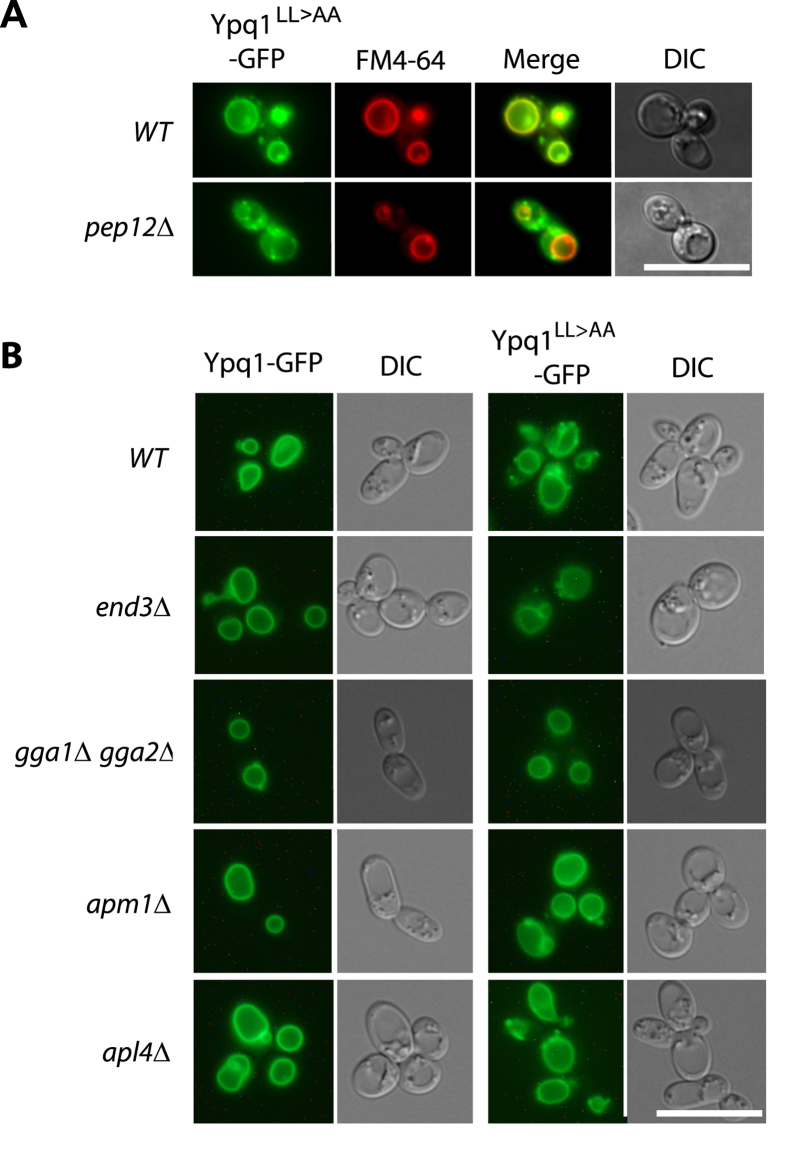
An acidic-dileucine motif promotes Ypq1 sorting to the ALP pathway. (**A**) Strains 23344c (*ura3*) and EN046 (*pep12∆ ura3*) transformed with the pLL034 (*YPQ1*^*LL>AA*^*-GFP URA3*) plasmid were grown on a glucose-ammonium medium. Cells were allowed to internalize FM4-64 for 15 min to label the vacuole before imaging. (**B**) Strains 23344c (*ura3*), LL115 (*end3∆ ura3*), JA445 (*gga1∆ gga2∆ ura3*), LL078 (*apm1∆ ura3*) and LL066 (*apl4∆ ura3*) transformed with the pLL063 (*YPQ1-GFP URA3*) or pLL034 (*YPQ1*^*LL>AA*^*-GFP URA3*) plasmids were grown on a glucose-ammonium medium. Cells were allowed to internalize FM4-64 for 15 min to label the vacuole before imaging. Scale bar: 10 μm.

AP-3-dependent sorting of transmembrane proteins is often mediated by tyrosine-based (YXXØ) or dileucine-based ([DE]XXXL[LI]) signals (where Ø is a bulky hydrophobic residue and X any amino acid)^15^. Ypq1 contains an EQQPLL sequence in its second large loop facing the cytosol. The dileucine of this motif is preceded by a proline, a feature observed in several cargoes using the ALP pathway^16^. A dileucine-to-dialanine substitution mutant of Ypq1 (Ypq1^LL>AA^) was found to be targeted to the vacuolar membrane, but also to decorate punctate structures (Fig. 3A). This phenotype resembles that observed for wild-type Ypq1 in AP-3-deficient cells, thus suggesting that Ypq1^LL>AA^ reaches the vacuole via the alternative CPY pathway. In support of this view, Ypq1^LL>AA^ produced in a *pep12*Δ mutant failed to be targeted to the vacuole, and was missorted into cytosolic punctate structures as observed for Ypq1 in the *apm3*Δ *pep12*Δ and *apl5*Δ *pep12*Δ mutants (Fig. 3A). These results show that the acidic dileucine motif of Ypq1 is required for its AP-3-dependent sorting to the vacuole, but not for its Pep12-dependent delivery to the vacuole. These conclusions are consistent with the results of a recent study published by S. Emr and coworkers during preparation of this manuscript^17^. We next investigated in more detail how the Ypq1^LL>AA^ variant traffics to the vacuole. We first envisaged the possibility that this mutant protein, because of its failure to use the ALP pathway, might first be missorted to the plasma membrane before undergoing rapid endocytosis and subsequent Pep12-dependent delivery to the vacuolar membrane. This model was not supported by our observations: the Ypq1^LL>AA^ did not accumulate at the cell surface in an *end3*Δ mutant defective in endocytosis, which indicates that it reaches the vacuole via the CPY pathway (Fig. 3B). We then hypothesized that delivery of Ypq1^LL>AA^ to the vacuole might involve its sorting from the Golgi to endosomes thanks to alternative adaptors such as the AP-1 complex or the monomeric GGA proteins. We expressed the Ypq1^LL>AA^ mutant protein in *gga1*Δ *gga2*Δ cells, lacking the redundant Gga1 and Gga2 adaptors, and in *amp1*Δ*, amp2*Δ and *apl4*Δ cells, lacking subunits of the AP-1 complex. In each of these mutants, the protein was found still to reach the vacuole (Fig. 3B). These observations suggest either that these adaptors act redundantly to promote sorting of Ypq1^LL>AA^ to the vacuole via the CPY pathway or that other adaptors are involved.

### Ypq2 and Ypq3 also traffic to the vacuole via the ALP pathway, but undergo different fates when the ALP pathway is nonfunctional

**Figure 4:**
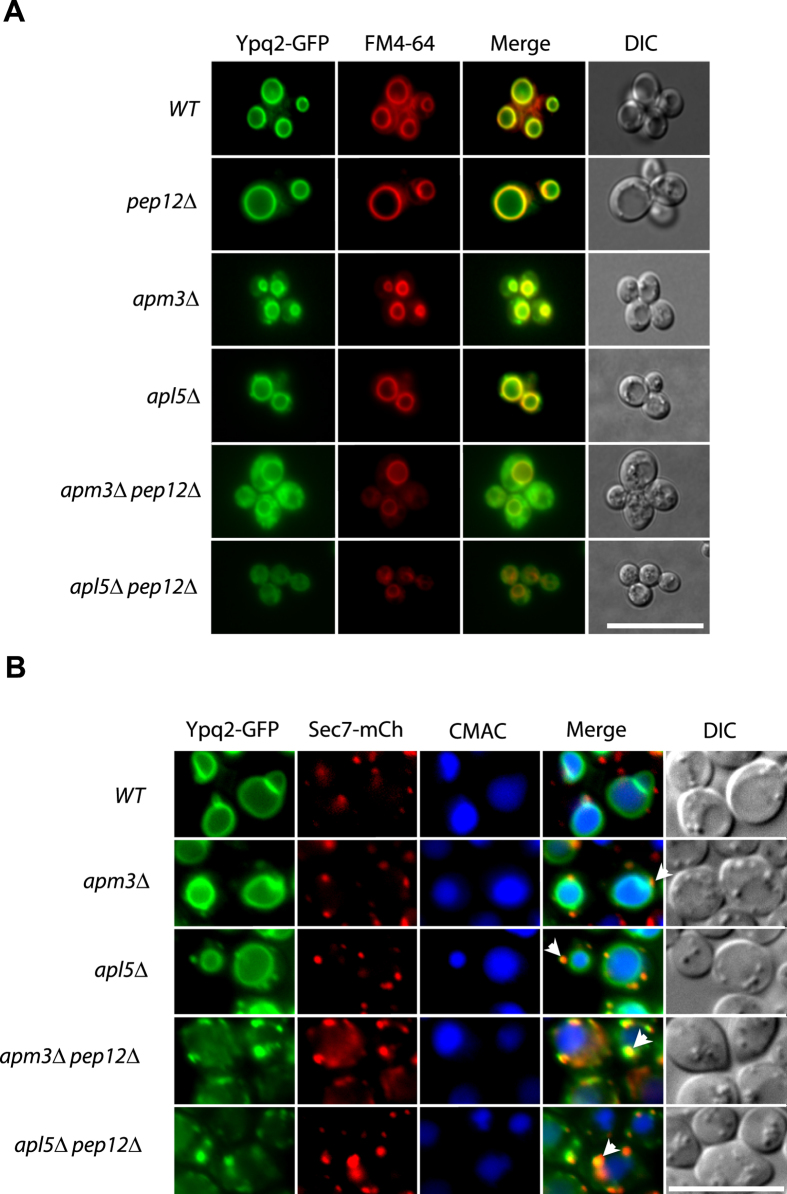
Ypq2 can reach the vacuolar membrane via the ALP or CPY pathways. **(A)** Strains 23344c (*ura3*), EN046 (*pep12∆ ura3*), LL057 (*apm3∆ ura3*), LL061 (*apl5∆ ura3*), LL088 (*apm3∆ pep12∆ ura3*) and LL041 (*apl5∆ pep12∆ ura3*) transformed with the pLL161 (*YPQ2-GFP URA3*) plasmid were grown on a glucose-ammonium medium. Cells were allowed to internalize FM4-64 for 15 min to label the vacuole before imaging. (**B**) Strains GC004 (*ura3 leu2*), BOA003 (*apm3∆ ura3 leu2*), BOA005 (*apl5∆ ura3 leu2*), BOA007 (*apm3∆ pep12∆ ura3 leu2*), BOA008 (*apl5∆ pep12∆ ura3*) transformed with the pLL161 (*YPQ2-GFP URA3*) or pBOA010 (*SEC7-mCherry LEU2)* plasmids were grown on a glucose-ammonium medium. Cells were allowed to internalize CMAC for 30 min to label the vacuolar lumen before imaging. Scale bar: 10 μm. A quantification of the sublocalization patterns of Ypq2-GFP (A) and Ypq1-GFP-Sec7-mCherry (**B**) can be found as Supplementary Figure S2 online.

The Ypq2 and Ypq3 proteins, very similar in sequence to Ypq1, also localize to the vacuolar membrane^6^ and both also contain an acidic dileucine in the second cytosolic loop. The results of Fig. 4A show that Ypq2 traffics normally to the vacuole in a *pep12*Δ mutant. This proved true also in *apm3*Δ and *apl5*Δ mutants defective in the ALP pathway, where it was additionally found to decorate punctate structures, a phenotype not observed in wild-type cells. A high proportion of these punctate structures were also labeled with Sec7-mCherry (Fig. 4B), indicating that Ypq2, similarly to Ypq1, tends to accumulate in the Golgi when the ALP pathway is deficient. In both *apm3*Δ *pep12*Δ and *apl5*Δ *pep12*Δ double mutants, Ypq2 largely mislocalized to small punctate cytosolic structures, many of which were decorated with Sec7-mCherry, although a fraction of the protein seemed properly delivered to the vacuole (Fig. 4A,B) (for a quantification of the sublocalization patterns, see Supplementary Fig. S2 online). These results indicate that Ypq2 behaves like Ypq1 in that it uses primarily the ALP pathway to reach the vacuole. They also show that when this pathway is defective, Ypq2 resides longer in the Golgi but can still be delivered to the vacuolar membrane, mainly via the CPY pathway.

**Figure 5:**
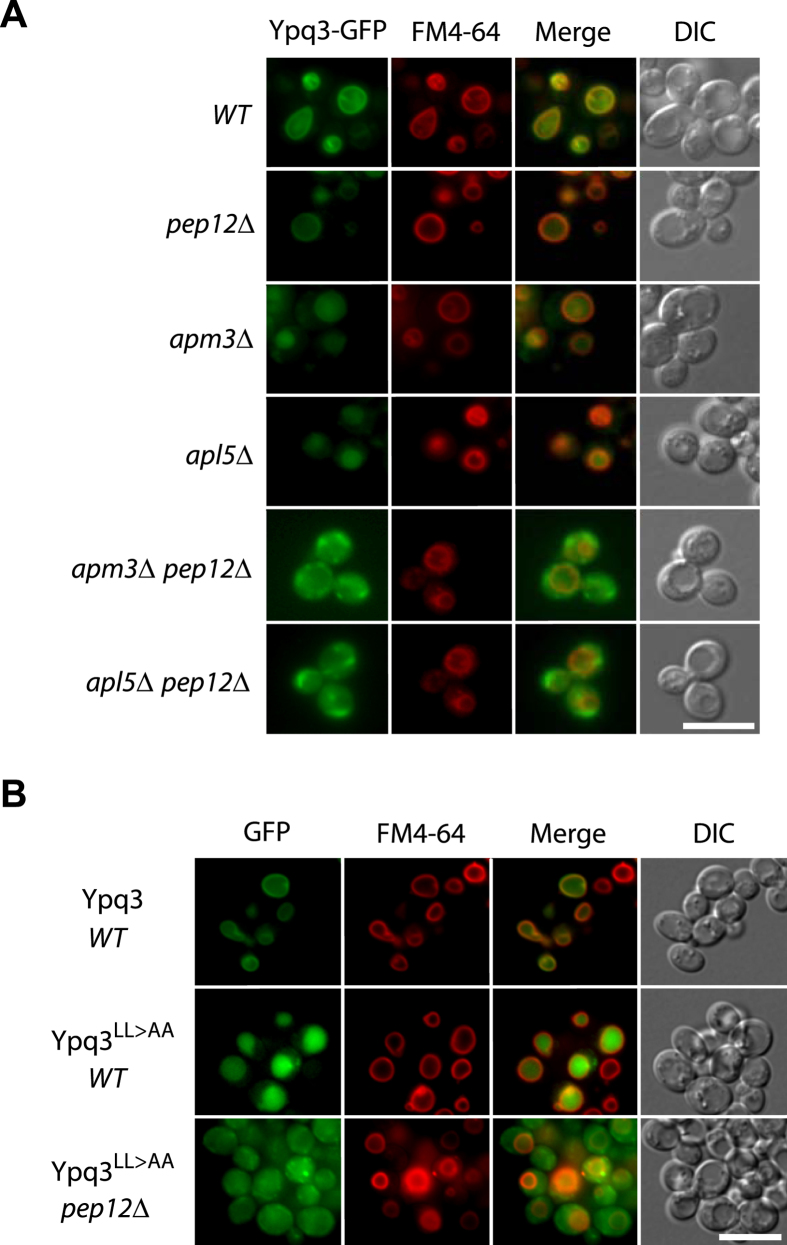
Ypq3 is delivered to the vacuolar membrane via the ALP pathway and is targeted to the vacuolar lumen if sorting via the ALP pathway is defective. (**A**) Strains 23344c (*ura3*), EN046 (*pep12∆ ura3*), LL057 (*apm3∆ ura3*), LL061 (*apl5∆ ura3*), LL088 (*apm3∆ pep12∆ ura3*) and LL041 (*apl5∆ pep12∆ ura3*) transformed with the pLL106 (*GAL1-YPQ3-GFP URA3*) plasmid were grown on raffinose-ammonium medium, galactose (3%) was added for 3 hours, and glucose (3%) was provided to cells for 2 hours. Cells were allowed to internalize FM4-64 for 15 min to label the vacuole before imaging. (**B**) Strain 23344c (*ura3*) transformed with the pLL106 (*GAL1-YPQ3-GFP URA3*) and pLL168 (*GAL1-YPQ3*^*LL>AA*^*-GFP URA3*) plasmids and strain EN046 (*pep12∆ ura3*) transformed with the pLL168 (*GAL1p-YPQ3*^*LL>AA*^*-GFP URA3*) plasmid were grown and analyzed as in (**A**). Scale bar: 5 μm. A quantification of the sublocalization patterns of Ypq3-GFP can be found as Supplementary Figure S4 online.

As expression of a *YPQ3-GFP* gene under the control of the natural promoter of *YPQ3* yielded a barely detectable level of Ypq3-GFP, we expressed the fusion gene under the control of a galactose-inducible promoter. To reduce the risk of mislocalization due to Ypq3-GFP overproduction, the cells were first grown on raffinose, then galactose was added for 3 hours, and finally glucose was provided for two hours to repress transcription of the *YPQ3-GFP* gene. This transiently induced Ypq3-GFP, which accumulated in the cell at a level close to the endogenous level of Ypq1-GFP (see Supplementary Fig. S3, online), was found to localize to the vacuolar membrane (Fig. 5A), in keeping with our previous results^6^. This vacuolar localization was also observed in the *pep12*Δ mutant. In the *apm3*Δ and *apl5*Δ mutants, however, Ypq3 was mainly missorted to the lumen of the vacuole. This missorting was dependent on Pep12, since Ypq3 failed to be targeted to the vacuole in *apm3*Δ *pep12*Δ and *apl5*Δ *pep12*Δ double mutants (Fig. 5A) (for a quantification of the sublocalization patterns, see Supplementary Fig. S4 online). These results suggest that, like Ypq1 and Ypq2, Ypq3 uses mainly the ALP pathway to reach the vacuolar membrane. When components of the AP-3 complex are lacking, Ypq3 is redirected in a Pep12-dependent manner to endosomes, where it is sorted into the multivesicular body pathway (MVB), resulting in its delivery to the vacuolar lumen. This interpretation was further assessed by isolating a Ypq3^LL>AA^ mutant that should not be recognized by the AP-3 adaptor complex. In wild-type cells the Ypq3^LL>AA^ variant was clearly missorted to the vacuolar lumen, but in a *pep12*Δ mutant it was redirected to small cytosolic punctate structures (Fig. 5B).

We considered that the different behavior of Ypq3 compared to Ypq1 and Ypq2 in AP-3 deficient strains might be due to the fact that Ypq3-GFP was synthesized in cells growing in the presence of galactose, or because transcription of the *YPQ3-GFP* gene was induced using the strong *GAL* promoter. This, however, seems unlikely because the Ypq1-GFP and Ypq2-GFP proteins transiently induced in wild-type and mutant strains using the same *GAL* promoter localized in cells as when they were expressed under their own gene promoters (see Supplementary Fig. S5 online). Furthermore, although the fluorescent signal was barely detectable, we obtained evidence that Ypq3-GFP expressed using the natural *YPQ3* gene’s promoter labeled the membrane of vacuoles in the wild-type strain, but not in the *apl5*Δ mutant, a phenotype clearly different from those obtained with Ypq1-GFP and Ypq2-GFP. In this mutant, Ypq3-GFP was present in punctate structures likely corresponding to the Golgi, and its missorting to the vacuolar lumen was not clearly visible, likely because the fluorescence was too weak (see Supplementary Fig. S6 online).

In conclusion, as shown above for Ypq1, the Ypq2 and Ypq3 proteins use mainly the ALP pathway to reach the vacuolar membrane and are deviated to endosomes when this pathway is defective. Yet while Ypq1 and Ypq2 transiting through endosomes reach the vacuolar membrane efficiently, Ypq3 is more prone to be sorted into the MVB pathway thus leading to its targeting to the vacuolar lumen.

### PQLC2 produced in yeast uses the ALP pathway and its dileucine motif to reach the vacuolar membrane

**Figure 6:**
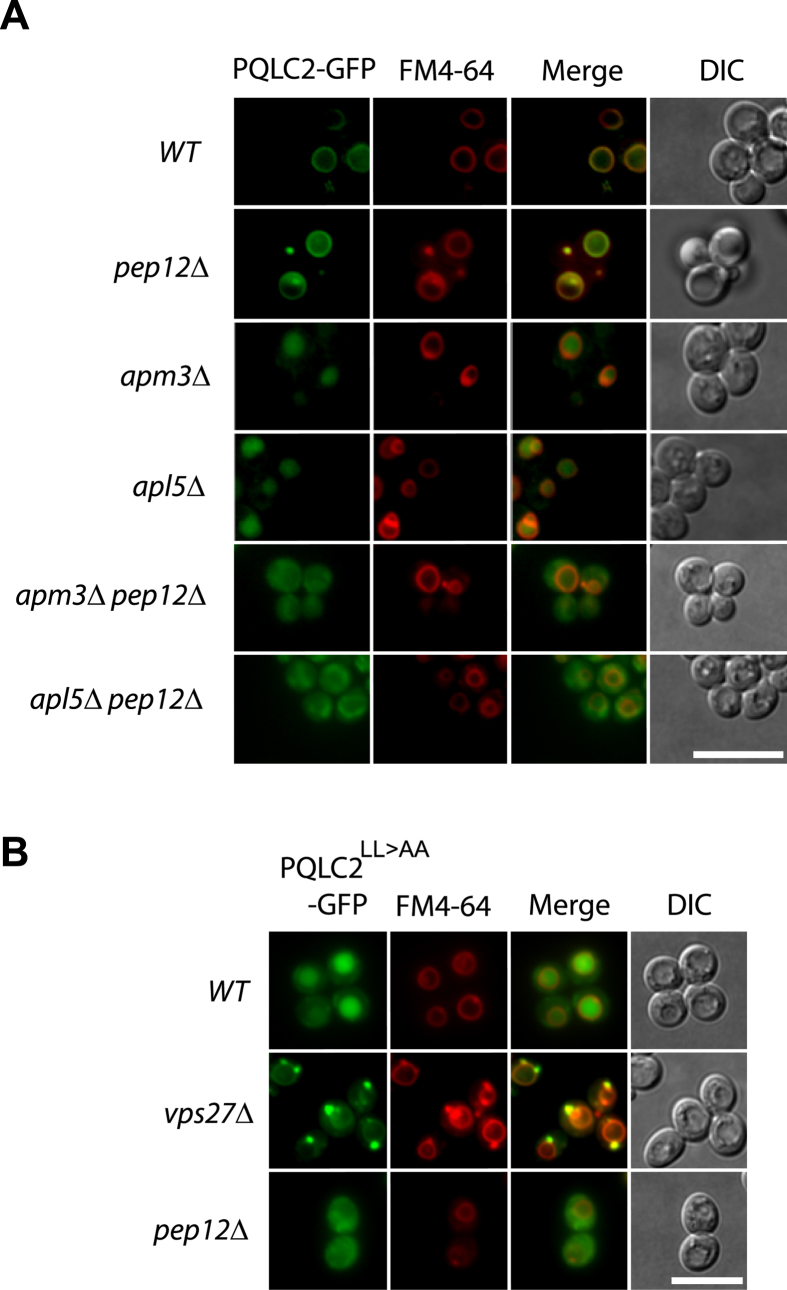
PQLC2 expressed in yeast is sorted to the vacuolar membrane via the ALP pathway. (**A**) Strains 23344c (*ura3*), EN046 (*pep12∆ ura3*), LL057 (*apm3∆ ura3*), LL061 (*apl5∆ ura3*), LL088 (*apm3∆ pep12∆ ura3*) and LL041 (*apl5∆ pep12∆ ura3*) transformed with the pCJ502 (*GAL1-rPQLC2-GFP URA3*) plasmid were grown and treated as in Fig. 5 before imaging. (**B**) Strains 23344c (*ura3*), JA770 (*vps27∆ ura3*) and EN046 (*pep12∆ ura3*) transformed with pBOA006 (*GAL1-rPQLC2*^*LL>AA*^*-GFP URA3*) plasmid were grown and treated as in Fig. 5 before imaging. Scale bar: 5 μm. A quantification of the sublocalization patterns of PQLC2-GFP can be found as Supplementary Figure S7 online.

In a previous study, rat PQLC2 was found to localize to lysosomes in HeLa cells, but its PQLC2^LL>AA^ mutant, in which the C-terminal dileucine is replaced with a dialanine, displayed a more diffuse distribution through the cell and was partially missorted to the plasma membrane^6^. Furthermore, PQLC2 produced in yeast was found to localize to the vacuolar membrane, where it is functional, since it was found to complement the growth phenotype of a *ypq2*Δ mutant^6^. The results in Fig. 6A show that PQLC2 was properly targeted to the yeast vacuole in a *pep12*Δ mutant, but deviated to the vacuolar lumen in *apm3*Δ and *apl5*Δ mutants. This targeting to the vacuolar lumen was impaired in *apm3*Δ *pep12*Δ and *apl5*Δ *pep12*Δ double mutants, where PQLC2 was found to be diffusely distributed through the cytosol (for a quantification of the sublocalization patterns, see Supplementary Fig. S7 online). We also expressed in wild-type and mutant yeast strains the PQLC2^LL>AA^ mutant. In wild-type yeast, PQLC2^LL>AA^ was found to localize to the vacuolar lumen, but it was found distributed throughout the cytosol in the *pep12*Δ mutant (Fig. 6B). Sorting of cargoes to the multivesicular body pathway is typically impaired in a *vps27*Δ mutant lacking a key component of the ESCRT-0 complex^18^. In the *vps27*Δ mutant, PQLC2^LL>AA^ was stacked in a large perivacuolar compartment: the class E compartment typically observed in this category of mutant (Fig. 6B). These results show that PQLC2 produced in yeast behaves like the endogenous Ypq3: it is sorted to the vacuole via the ALP pathway in a manner dependent on proper recognition of its dileucine motif by the AP-3 adaptor complex. When this recognition is impaired because the dileucine motif is mutated or a component of the AP-3 complex is lacking, PQLC2 is deviated to endosomes, where it is efficiently sorted into the MVB pathway, finally reaching the vacuolar lumen.

### Depletion of an AP-3 subunit impairs delivery of PQLC2 to lysosomes

**Figure 7:**
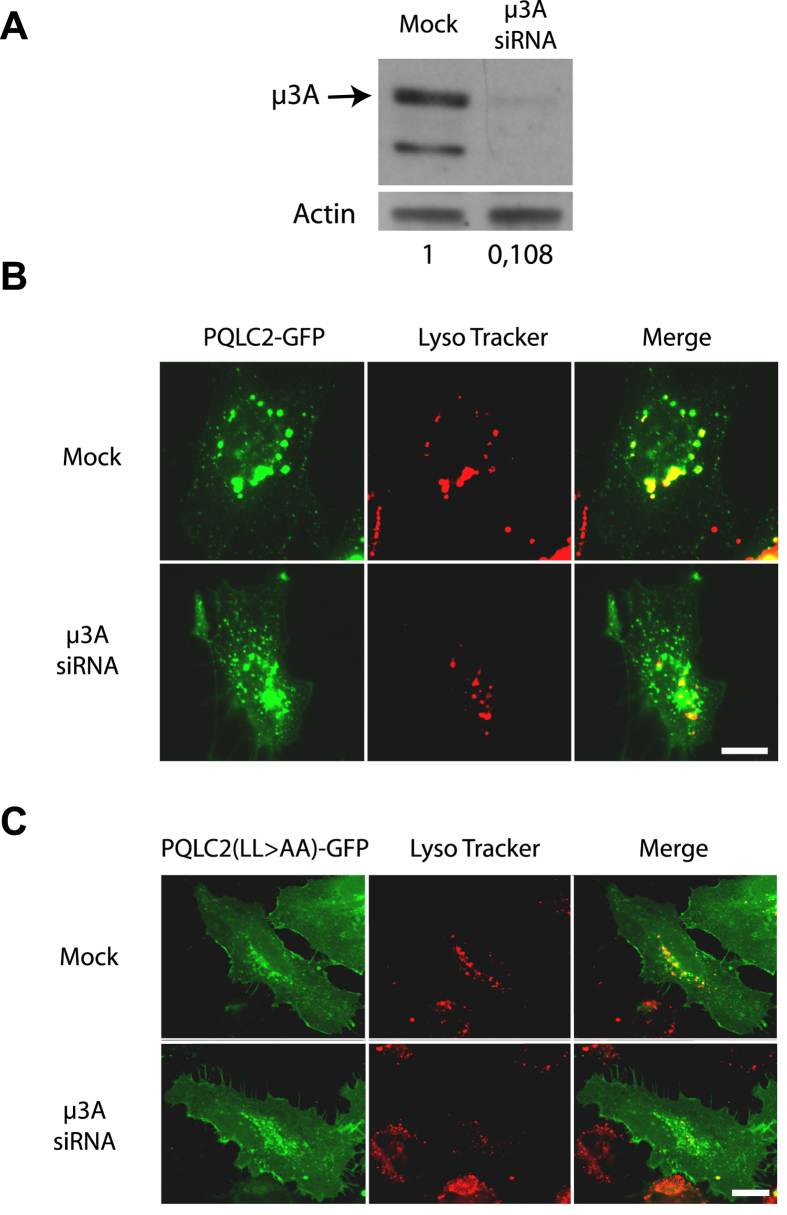
The AP-3 adaptor complex is required for localization of PQLC2 to the lysosome in HeLa cells. (**A**) HeLa cells were transfected three times with siRNAs directed to the μ3A subunit of the AP3 complex. 48 hours after the third round of transfection, equivalent amounts of homogenates of mock and siRNA-treated cells were subjected to SDS-PAGE and immunoblotting using an antibody against the μ3A subunit of the AP3 complex. Numbers correspond to the relative levels of the μ3A subunit estimated using actin as a reference (**B**) At the third round of transfection with siRNAs, cells were cotransfected with the *rPQLC2-EGFP* or *rPQLC2*^*LL>AA*^*-EGFP* plasmids. After 48 hours, cells were incubated for 2 hours with of the Lysotracker-Red DND-99 and fixed before imaging. Scale bar: 10 μm.

**Figure 8:**
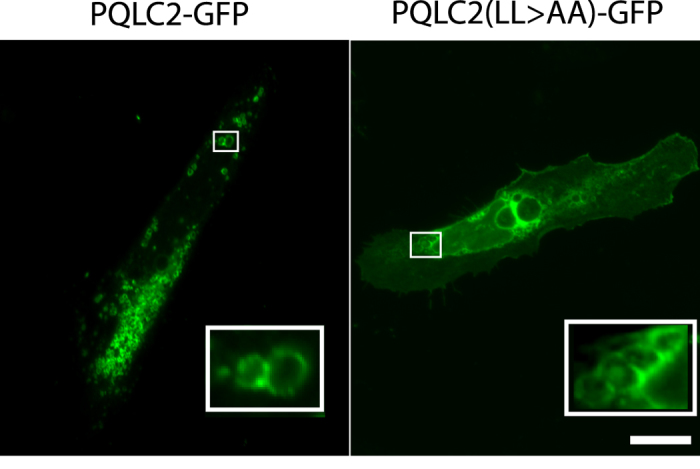
PQLC2 is not delivered to the lumen of lysosomes when sorting via the AP-3 complex is impaired. HeLa cells transfected with the *PQLC2-EGFP* or *PQLC2*^*LL>AA*^*-EGFP* plasmids were treated with vacuolin-1 for 2 hours. Cells were fixed and examined by fluorescence microscopy. Scale bar: 10 μm.

PQLC2-GFP produced in HeLa cells largely colocalizes with the LAMP1 lysosomal marker. Localization of PQLC2 to the lysosomal membrane was further supported by semiquantitative mass spectrometry analysis of proteins in preparations highly enriched in lysosomal membranes from rat liver cells^6^. To determine if the AP-3 adaptor contributes to sorting of PQLC2 to the lysosomes, we used small interfering RNA (siRNA) to inhibit synthesis of the μ3A subunit of AP-3 in HeLa cells. Immunoblot analysis confirmed that the two typical bands corresponding to μ3A subunits^19^ were barely detectable in μ3A-siRNA-treated cells, in contrast to control cells (Fig. 7A). This depletion of μ3A strongly perturbed the localization of PQLC2-GFP (Fig. 7B): the protein localized largely to numerous intracellular punctate structures not labeled by the Lyso Tracker dye accumulating in lysosomes, and was also found at the cell surface, including the microvilli, indicating that part of the protein was also misrouted to the plasma membrane. This contrasts with the localization of PQLC2 in mock-treated cells, where the protein was present in punctate structures also strongly labeled by the Lyso Tracker dye, as expected. These results suggest that the AP-3 complex contributes to proper localization of PQLC2 to lysosomes. We also investigated the localization of the PQLC2^LL>AA^ mutant (Fig. 7C). In control HeLa cells, PQLC2^LL>AA^ was found distributed in intracellular punctate structures not or very poorly labeled by the Lyso Tracker dye, and it was also clearly present at the cell surface, in keeping with previous observations^6^. This localization was not significantly perturbed in μ3A-siRNA-treated cells, supporting the view that the role of the dileucine motif of PQLC2 is to mediate intracellular traffic via the AP-3 complex. We next examined whether PQLC2^LL>AA^ is rerouted to the lumen of endosomal/lysosomal compartments, as when it is produced in yeast. HeLa cells expressing PQLC2-GFP were treated for two hours with vacuolin-1, a compound inducing homotypic fusion of compartments of the endosomal/lysosomal system, resulting in formation of large, swollen structures (Fig. 8). PQLC2-EGFP was found to localize, as expected, to the limiting membrane of these enlarged endosomal/lysosomal compartments. The same was true in cells producing PQLC2^LL>AA^, suggesting that the mutant protein is not efficiently sorted into the MVB pathway. In conclusion, the AP-3 adaptor complex seems to play an important role in targeting PQLC2 to the lysosome, most likely via recognition of its C-terminal dileucine. When this recognition is impaired, the protein is deviated to the cell surface and to the limiting membrane of internal compartments.

## Discussion

Integral membrane proteins of lysosomes in animal cells, of tonoplasts in plant cells, and of the vacuole in yeast follow the secretory pathway and are sorted from the Golgi to the destination membrane after packaging into vesicles. A crucial step in this sorting is recognition, by specialized adaptor proteins or complexes^15^, of the dileucine- or tyrosine-based motif typically harbored by the proteins to be sorted. Previous work in yeast has highlighted the role of the AP-3 complex in the vacuolar targeting of several transmembrane proteins, including alkaline phosphatase (Pho8/ALP)^20^,^21^,^22^, the Vma3 SNARE required for vacuole fusion^16^,^20^, casein kinase 3 (Yck3)^23^, two proteins acting as putative adaptors for protein ubiquitylation (Sna2 and −4)^24^,^25^, the Ncr1 protein involved in sphingolipid metabolism^26^, and a protein involved in autophagy (Atg27)^27^. To date, however, the trafficking of yeast vacuolar transporters has been little investigated. Here we report that the Ypq transporters proposed to mediate export of basic amino acids stored in the vacuole^6^ are also targeted to the vacuole in a manner dependent on the AP-3 adaptor complex. Our data suggest that this complex recognizes an acidic dileucine motif (DxxLL) present in the second cytosolic loop of each of these proteins. We find that when this dileucine motif is mutated or the AP-3 complex is nonfunctional, the Ypq proteins tend to accumulate in the Golgi but are also rerouted to endosomes. The Ypq1 and Ypq2 proteins reaching the endosomes are efficiently delivered to the vacuolar membrane. In contrast, the Ypq3 transporter tends to be missorted to the vacuolar lumen, indicating that Ypq3 deviated to endosomes is efficiently sorted via the MVB pathway into vesicles budding into the endosomal lumen, probably because Ypq3 undergoes ubiquitylation^28^. How Ypq proteins that fail to use the ALP pathway are rerouted to endosomes remains undetermined. This sorting likely involves their packaging into vesicles thanks to alternative adaptors, e.g. the AP-1 complex and/or the monomeric GGA proteins. Yet we have failed to show a specific role of these adaptors in targeting the Ypq1^LL>AA^ mutant protein to the vacuole. This suggests that these adaptors function redundantly or that others promote this sorting to endosomes. Previous work has shown that an acidic dileucine promotes AP-3-dependent sorting of the Pho8, Vam3, and Sna4 proteins^24^,^29^ to the vacuole, whereas a tyrosine-based motif plays a similar role for vacuolar targeting of Sna2, Yck3, and Atg27^23^,^25^,^27^. Interestingly, the Sna2 protein contains a second tyrosine-based motif promoting AP-1-dependent sorting to the vacuole^25^. When the interaction with AP-3 is impaired, these proteins undergo different fates: proper sorting to the vacuole via the alternative CPY pathway^20^,^21^,^26^, missorting to the vacuolar lumen via the MVB pathway^24^, or rerouting to the plasma membrane^23^. Studies on plant cells have likewise revealed an important role of the AP-3 complex in sorting membrane proteins (including transporters) to the tonoplast^30^. In animal and human cells, the AP-3 adaptor is known to promote delivery of several lysosomal membrane proteins, including LAMP-1, LIMP-2, and CD63^31^,^32^. The AP-3 complex also mediates sorting of several proteins (eg. tyrosinase and OCA2, a protein similar to transporters) to the membrane of the melanosome, a lysosome-related organelle of melanocytes and ocular pigment cells in which melanin pigments are synthesized and stored^33^,^34^. We now report that the PQLC2 transporter also needs the AP-3 adaptor complex to be properly targeted to lysosomes in HeLa cells, as well as to the vacuole when produced in yeast. A recent study has shown that sorting to the lysosome of cystinosin, the cystine exporter of the PQ-loop family, involves a tyrosine-based motif interacting with the AP-3 complex. If this interaction is impaired, cystinosin tends to be targeted to the plasma membrane^35^. Furthermore, when produced in yeast, cystinosin is delivered to the vacuolar membrane but is partially deviated to the cell surface if the AP-3 complex is deficient (our unpublished data). These data further support the conclusion that yeast and mammalian PQ-loop transporters traffic to the vacuole or lysosome via similar AP-3-dependent mechanisms. However, the AP-3 complex of yeast is thought to recruit cargoes only at the trans-Golgi for direct targeting to the vacuole whereas that of mammalian cells operates at early endosomes where it appears to sort proteins to deliver them to late endosomes, lysosomes or lysosome-related organelles, ie. late-maturation-stage compartments where formation of intraluminal vesicles via the multivesicular body pathway is no longer active. The basic role of AP-3 in yeast and mammalian cells could therefore be to circumvent possible delivery of cargoes to the lumen of these compartments^14^, a model supported by our observation that Ypq3 and PQLC2 produced in yeast are targeted to the vacuolar lumen when AP-3-mediating sorting is defective. Interestingly, a recent study reported that the Ypq1 transporter present at the vacuolar membrane can subsequently be targeted to the vacuolar lumen. This occurs under lysine-starvation conditions and requires prior ubiquitin-dependent sorting of Ypq1 to endosomes where the transporter is sorted into intraluminal vesicles^17^. It will thus be interesting to determine whether other lysosomal transporters unrelated to the PQ-loop family also use a dileucine- or tyrosine-based motif recognized by the AP-3 complex to reach their destination membrane circumventing the multivesicular body pathway, and if they can subsequently be targeted to the lumen of the lysosome via a pathway similar to the one described for Ypq1 in yeast.

## Methods

### Yeast strains, media, and plasmids

All *Saccharomyces cerevisiae* strains used in this study (Table 1) derive from the Σ1278b wild type. Cells were grown at 29°C in minimal buffered medium, pH 6.1^36^. In experiments for visualizing Ypq1-GFP or Ypq2-GFP produced from a gene expressed under the control of its own promoter, the carbon source was glucose (3%). The *YPQ3-GFP* and *PQLC2-GFP* fusion genes were expressed under the control of the galactose-inducible *GAL1* promoter. To transiently induce their expression, cells were first grown on raffinose (3%) and glucose (0.3%), the galactose (3%) was added for 3 hours, and finally glucose (3%) was provided to the cells 2 hours before their visualization. In all experiments, (NH_4_)_2_SO_4_ (10 mM) was the sole nitrogen source of the medium. The plasmids used in this work are listed in Table 2. The functionality of the Sec7-mCherry construct was verified by testing its capacity to complement a thermosensitive *sec7* mutant (see Supplementary Fig. S8, online). Experimental details, including the sequences of the oligonucleotides used in PCR reactions for the isolation of yeast mutant strains and plasmids, are available upon request.

### Fluorescence microscopy analysis of yeast cells

Subcellular localization of GFP-fused Ypq and PQLC2 was performed in yeast cells growing exponentially in liquid medium. Labeling of the vacuolar membrane with FM4-64 was performed as described previously^37^. In cells expressing the Sec7-mCherry protein, the vacuole was labeled with CMAC (7-amino-4-chloromethylcoumarin). Cells were deposited on a thin layer of 1% agarose and viewed at room temperature with a fluorescence microscope (Eclipse E600; Nikon) equipped with a 100x differential interference contrast NA 1.40 Plan-Apochromat objective (Nikon) and appropriate fluorescence light filter sets. Images were captured with a digital camera (DXM1200; Nikon) and ACT-1 acquisition software (Nikon). Final images were prepared with Photoshop CS (Adobe Systems). Quantification of the subcellular location patterns of Ypq-GFP proteins was based on images obtained in three independent experiments. For each experiment, one hundred randomly-selected cells were examined by eye and classified in different subcellular localization patterns: vacuolar membrane, vacuolar membrane and several dots, cytoplasmic distribution and dots, a combination of cytoplasmic distribution, dots and vacuolar membrane, and vacuolar lumen and dots.

### HeLa cell cultures, transfection, and fluorescence microscopy

HeLa cells were grown at 37 °C and 5% CO_2_ in DMEM-Glutamax (Life technologies) medium supplemented with 10% fetal bovine serum (Life technologies), 1% penicillin/streptomycin (Life technologies) and HEPES. To locate PQLC2-EGFP, cells were seeded at 5×10^4^ per well in 24-well dishes on coverslips. The cells were transfected with 0.3 μg of the relevant PQLC2-EGFP construct by treatment with lipofectamine 2000 reagent according to the manufacturer’s instructions. After 48 hours, the cells were incubated for 2 h with Lysotracker-Red DND-99 (250 nM, Life Technologies) or vacuolin-1 (5 μM, Chembridge). They were fixed for 20 min with 3% paraformaldehyde (PFA) (Merck) in phosphate-buffered saline (PBS) and mounted onto glass slides.

### RNA-mediated interference (RNAi)

RNAi was used to inhibit synthesis of the μ3A (AP3M1) subunit of the AP complex. It was performed with siRNA (QIAGEN, Valencia, CA) targeting the human sequence GGAUAGCCCUUACACUCAUTT. AllStars negative control siRNA was used as a control (mock). Cells were transfected with the relevant siRNA three times at 24-h intervals (20 nM) by means of lipofectamine 2000 (Life technologies) used according to the manufacturer’s protocol. The cells were analyzed 48 h after the third round of transfection.

### Protein extracts and western blotting

Cells were seeded at 1.5×10^6^ per well in 6-well dishes. 48 hours after the third round of transfection with siRNA, the cells were lysed with Nonidet-P40 buffer (50 mM Tris pH 8.0, 120 mM NaCl, 0,5% NP40) in the presence of protease inhibitors. The samples were then boiled in sample buffer, run on an SDS polyacrylamide gel, and subjected to western blotting. The blot was probed with anti-AP3M1 antibody (Abcam, ab113104).

## Additional Information

**How to cite this article**: Llinares, E. *et al.* The AP-3 adaptor complex mediates sorting of yeast and mammalian PQ-loop-family basic amino acid transporters to the vacuolar/lysosomal membrane. *Sci. Rep.* **5**, 16665; doi: 10.1038/srep16665 (2015).

## Supplementary Material

Supplementary Information

## Figures and Tables

**Figure 1:**
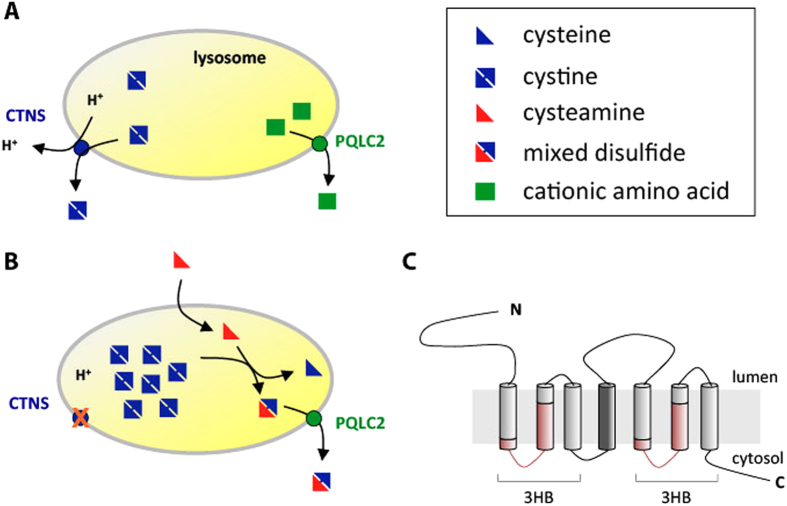
Role of the lysosomal cystinosin (CTNS) and PQLC2 transporters in normal and cystinotic cells. (**A**) In normal cells, CTNS catalyzes H^+^-coupled export of cystine and PQLC2 exports basic amino acids. (**B**) In cells of patients suffering of cystinosis, cystinosin is not functional, causing lysosomal accumulation of cystine. Cysteamine, the aminothiol drug used to treat cystinotic patients, enters the lysosome and reacts with cysteine to generate cysteine and the mixed disulfide cysteamine-cysteine, similar to lysine. The latter is exported from the lysosome via PQLC2. (**C**) Putative topology of PQLC2. The transmembrane domain connecting the duplicated triple helix bundles (3HB) is coloured in dark grey. The conserved PQ-loop motifs are coloured in red.

**Table 1:** Yeast strains used in this study.

Strain	Genotype	Source
23344c	*ura3*	Lab collection
EN046	*pep12*Δ *ura3*	Lab collection
LL061	*apl5*Δ *ura3*	This study
LL057	*apm3*Δ *ura3*	This study
LL088	*apm3*Δ *pep12*Δ *ura3*	This study
LL041	*apl5*Δ *pep12*Δ *ura3*	This study
BOA003	*apm3*Δ *ura3 leu2*	This study
BOA007	*apm3*Δ *pep12*Δ *ura3 leu2*	This study
BOA008	*apl5*Δ *pep12*Δ *ura3 leu2*	This study
BOA005	*apl5*Δ *ura3 leu2*	This study
LL115	*end3*Δ *ura3*	This study
JA445	*gga1*Δ *gga2*Δ *ura3*	This study
LL078	*apm1*Δ *ura3*	This study
LL066	*apl4*Δ *ura3*	This study
JA770	*vps27*Δ *ura3*	Lab collection
SEY5076	*MAT sec7-1 ura3-52 leu2-3 leu2-112 SUC2*	(38)
CG004	*ura3 leu2*	Lab collection

**Table 2:** Plasmids used in this study.

Plasmid	Description	Reference
pFL38	*CEN-ARS (URA3)*	(39)
p416 GAL1	*CEN-ARS-GAL1 (URA3)*	(40)
pLL063	*CEN-ARS YPQ1-GFP (URA3)*	(6)
pLL034	*CEN-ARS YPQ1(L128A, L129A)-GFP (URA3)*	This study
pLL161	*CEN-ARS YPQ2-GFP (URA3)*	(6)
pLL111	*CEN-ARS YPQ3-GFP (URA3)*	This study
pLL106	*CEN-ARS GAL1-YPQ3-GFP (URA3)*	(6)
pLL168	*CEN-ARS GAL1-YPQ3(L135A, L136A)-GFP (URA3)*	This study
pCJ502	*CEN-ARS GAL1-rPQLC2-GFP (URA3)*	(6)
pBOA006	*CEN-ARS GAL1-rPQLC2(L290A, L291A)-GFP (URA3)*	This study
pLL088	*CEN-ARS GAL1-YPQ1-GFP (URA3)*	This study
pCJ127	*CEN-ARS GAL1-YPQ2-GFP (URA3)*	This study
pFL36	*CEN-ARS (LEU2)*	(39)
pBOA010	*CEN-ARS SEC7-mCherry (LEU2)*	This study
