# Supplementary material for: The AP-3 adaptor complex mediates sorting of yeast and mammalian PQ-loop-family basic amino acid transporters to the vacuolar/lysosomal membrane
Source: Sci Rep. 2015 Nov 18;5:16665. doi: 10.1038/srep16665 (PMC4649669; doi:10.1038/srep16665)
Supplement: Supplementary Information [file srep16665-s1.pdf]

# Supplementary information

# The AP-3 adaptor complex mediates sorting of yeast and mammalian PQ-loop-family basic amino acid transporters to the vacuolar/lysosomal membrane

**Elisa Llinares, Abdoulaye Oury Barry, and Bruno André**

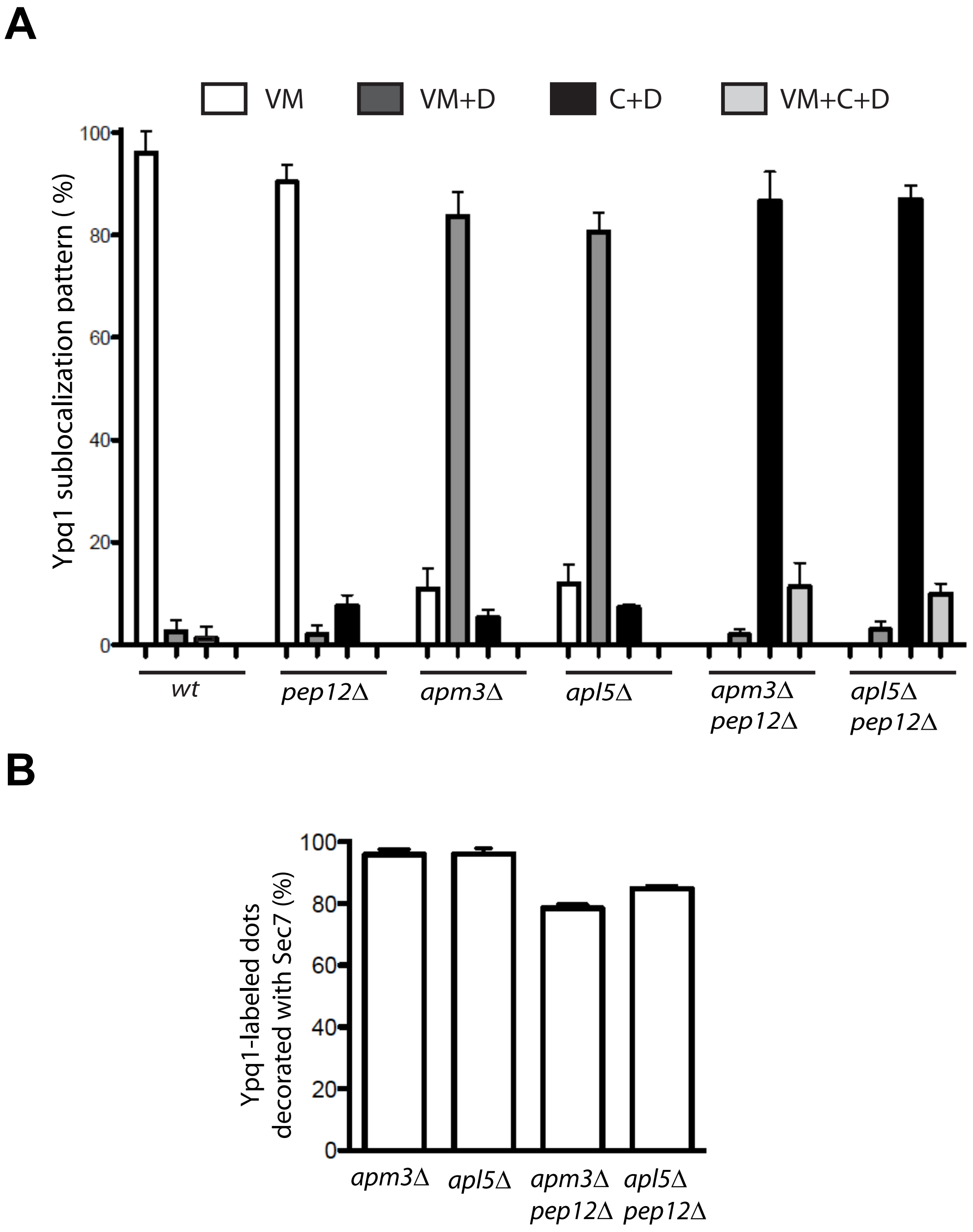


**Figure S1. Quantification of the subcellular localization patterns of Ypq1-GFP (see representative images in Fig. 2B, C).** (A) The subcellular localization of Ypq1-GFP was examined as described in Methods and classified into four patterns: vacuolar membrane (VM), vacuolar membrane + several dots (VM+D), cytoplasmic distribution + several dots (C+D), and vacuolar membrane + cytoplasmic distribution + several dots (VM+C+D). (B) Quantification of intracellular dots where Ypq1-GFP and Sec7-mCherry co-localized. The quantification was based on examination by eye of 100 cells. The wild-type was not examined as cells with several well visible dots were too rare. The error bars represent the standard deviation of three biological replicates.

**A**

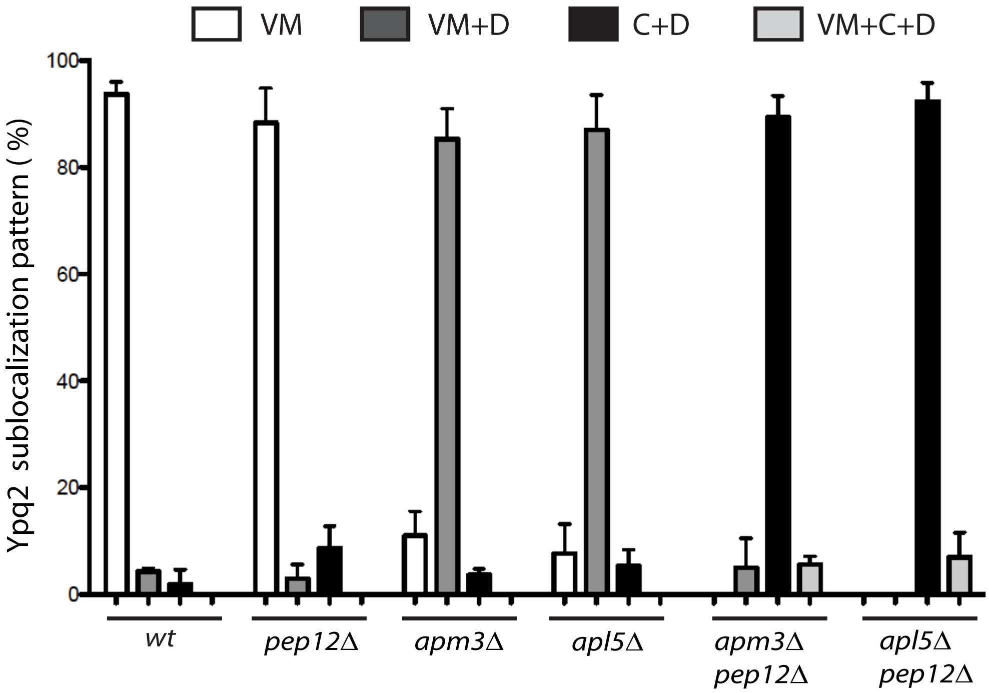


**B**

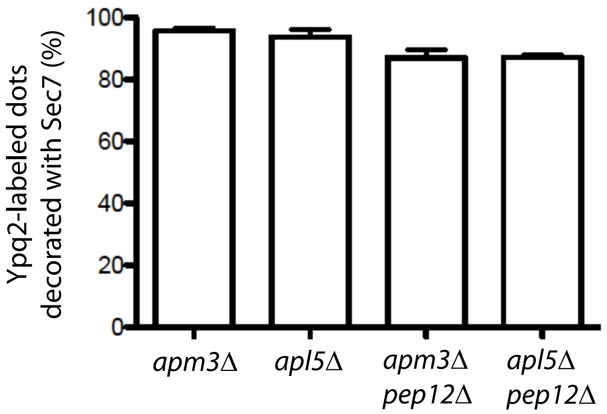


**Figure S2. Quantification of the subcellular localization patterns of Ypq2-GFP (see representative images in Fig. 4A, B).** (A) The subcellular localization of Ypq2-GFP was examined as described in Methods and classified into four patterns: vacuolar membrane (VM), vacuolar membrane + several well visible dots (VM+D), cytoplasmic distribution + several dots (C+D), and vacuolar membrane + cytoplasmic distribution + several dots (VM+C+D). (B) Quantification of intracellular dots where Ypq2-GFP and Sec7-mCherry co-localized. The quantification was based on examination by eye of 100 cells. The wild-type was not examined as cells with several well visible dots were too rare. The error bars represent the standard deviation of three biological replicates.

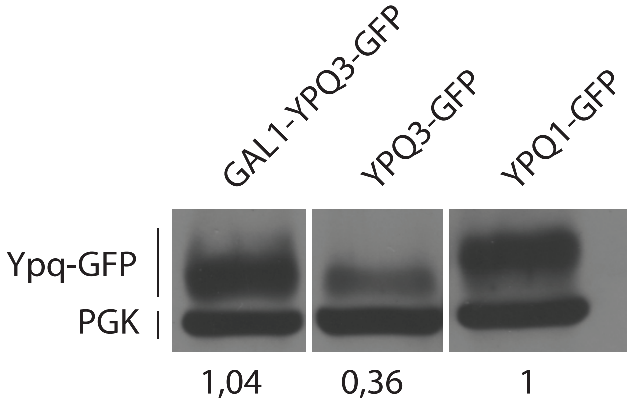


**Figure S3. The Ypq3-GFP protein, after transient induction of its gene by galactose, accumulates in cells at a level close to that of Ypq1-GFP expressed behind its own gene's promoter.** Strain 23344c (*ura3*) transformed with the pLL063 (*YPQ1-GFP URA3*) or pLL111 (*YPQ3-GFP URA3*) plasmids was grown on glucose medium, and the same strain transformed with pLL106 (*GAL1-YPQ3-GFP URA3*) plasmid was first grown on raffinose, galactose was added to the medium for three hours, and glucose was then added for two hours. Cell extracts were subjected to SDS-PAGE and immunoblotted against GFP or PGK. The intensity of the bands was measured using image J and normalization was based on the PGK signal. The intensity of the Ypq1-GFP band served as a reference to estimate the relative levels of Ypq3-GFP.

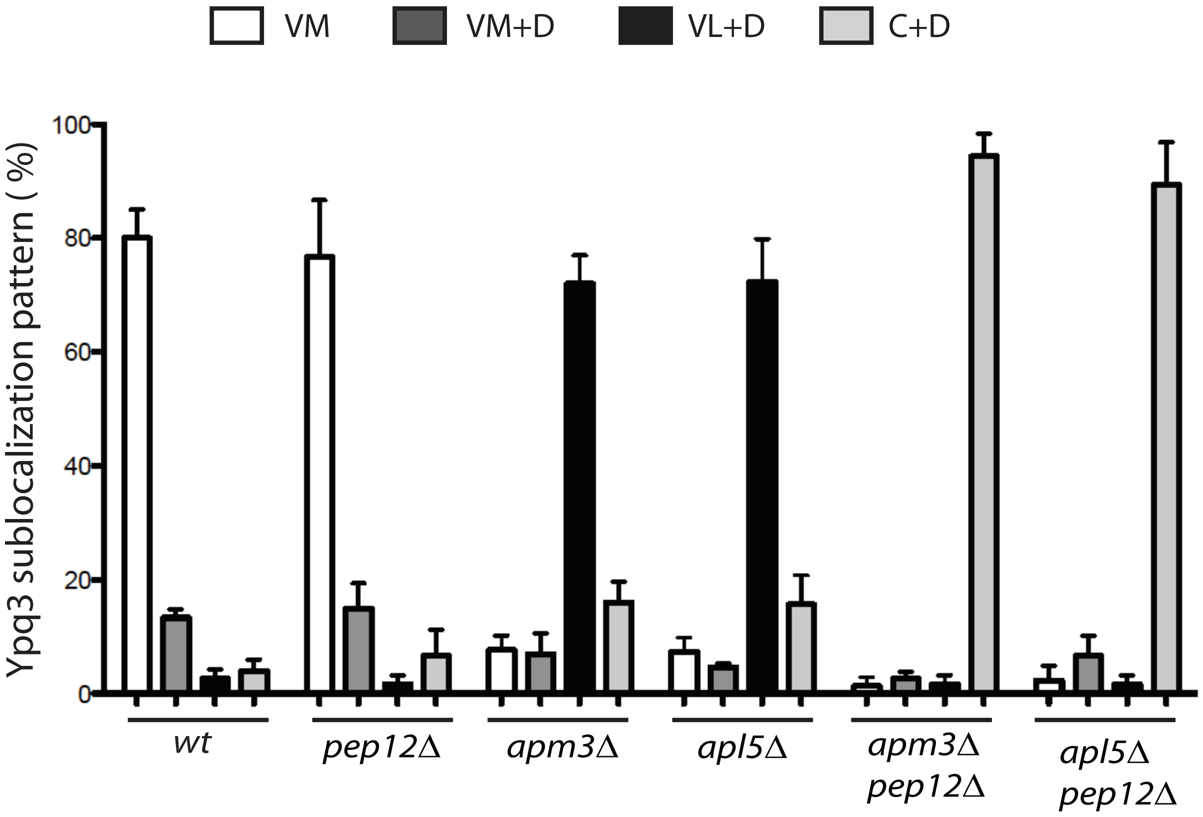


**Figure S4. Quantification of the subcellular localization patterns of Ypq3-GFP (see representative images in Fig. 5A).** The subcellular localization of Ypq3-GFP was examined as described in Methods and classified into four patterns: vacuolar membrane (VM), vacuolar membrane + several dots (VM+D), vacuolar lumen + several dots (VL+D), and cytoplasmic distribution + several dots (C+D). The error bars represent the standard deviation of three biological replicates.

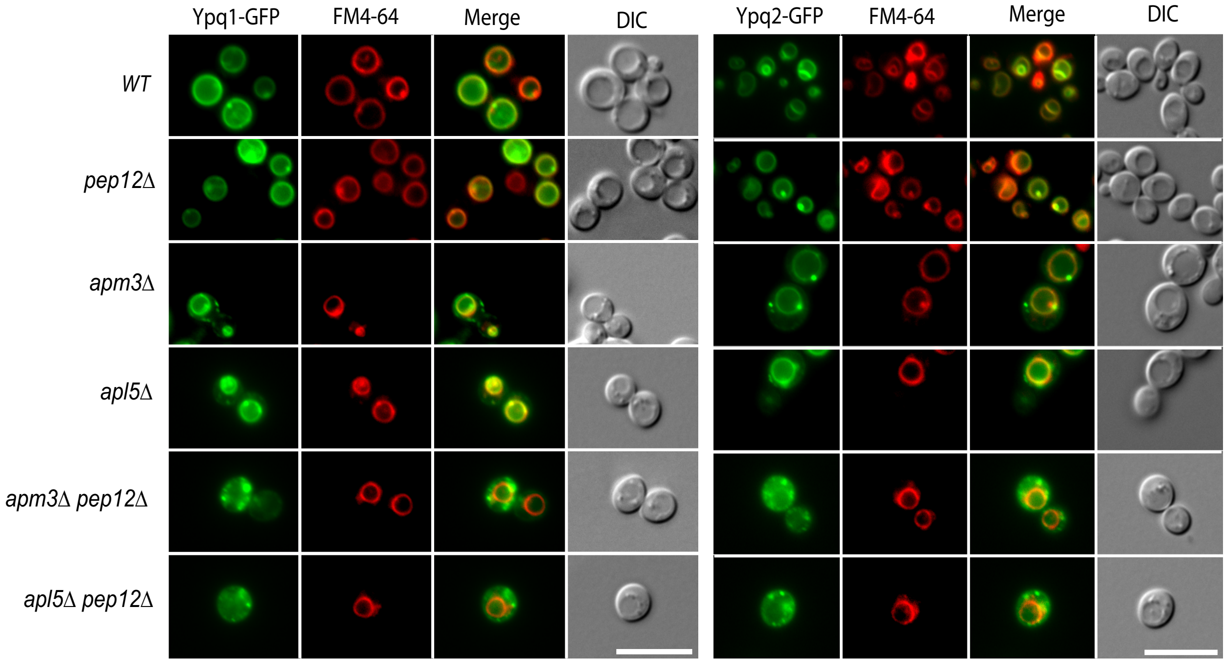


**Figure S5. Ypq1 and Ypq2 transiently produced in cells using the strong *GAL* promoter can reach the vacuolar membrane via the ALP or CPY pathways.** Strains 23344c (*ura3*), EN046 (*pep12Δ ura3*), LL057 (*apm3Δ ura3*), LL061 (*apl5Δ ura3*), LL088 (*apm3Δ pep12Δ ura3*) and LL041 (*apl5Δ pep12Δ ura3*) transformed with the pLL088 (*GAL1-YPQ2-GFP URA3*) or pLL088 (*GAL1-YPQ1-GFP URA3*) plasmids were grown on raffinose-ammonium medium, galactose (3%) was added for 3 hours, and glucose (3%) was provided to cells for 2 hours. Cells were allowed to internalize FM4-64 for 15 min to label the vacuole before imaging. Scale bar: 10µm.

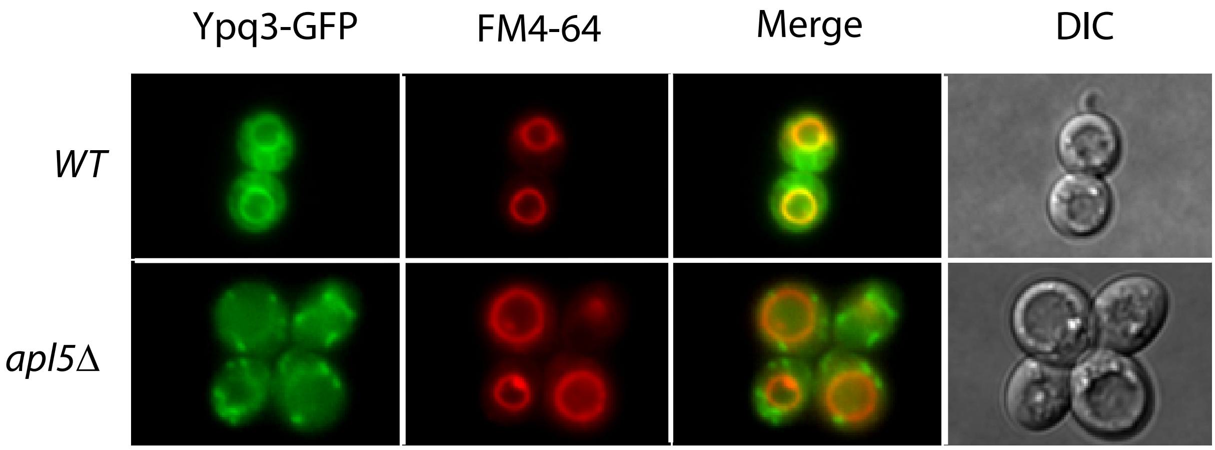


**Figure S6. Ypq3 expressed behind its own gene's promoter fails to reach the vacuolar membrane in AP-3 deficient cells.** Strains 23344c (*ura3*) and LL061 (*apl5*Δ *ura3*) transformed with pLL111 (*YPQ3-GFP URA3*) plasmid were grown on a glucose-ammonium medium. Cells were allowed to internalize FM4-64 for 15 min to label the vacuole before imaging.

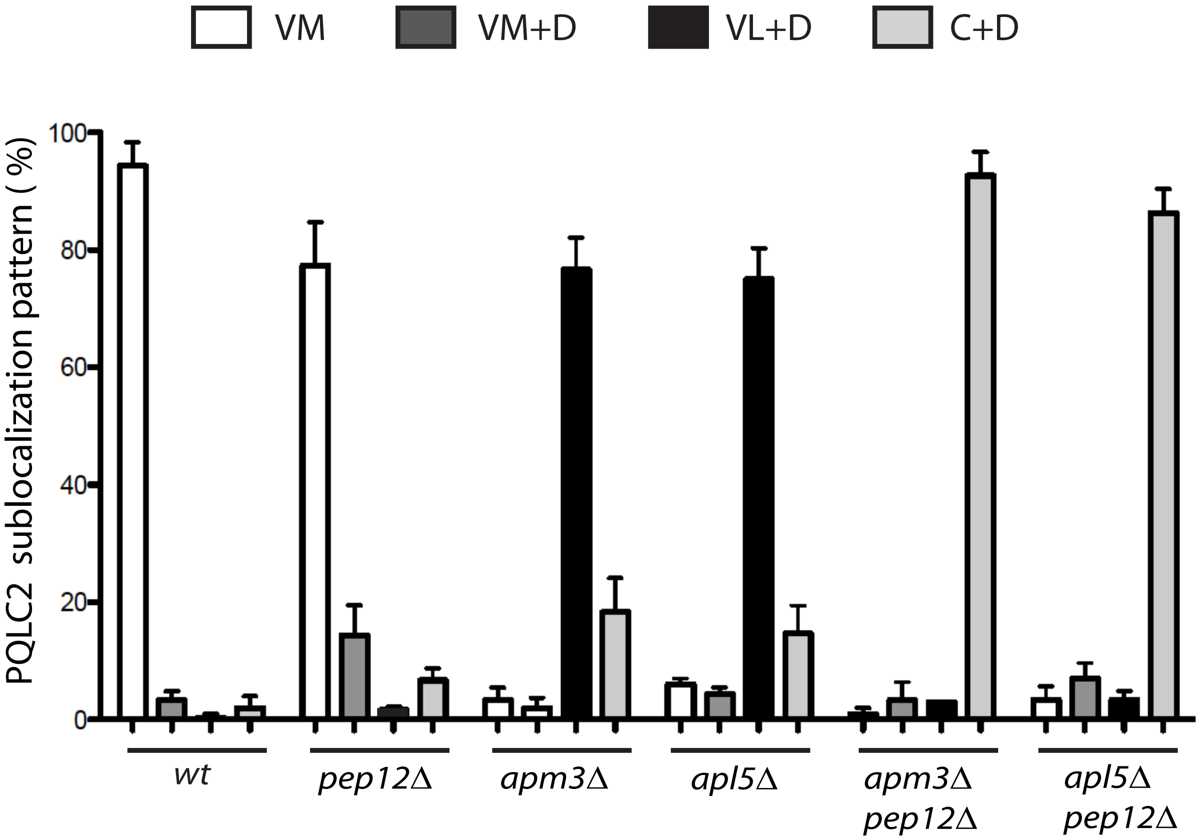


**Figure S7. Quantification of the subcellular localization patterns of PQLC2-GFP expressed in yeast (see representative images in Fig. 6A).** The subcellular localization of PQLC2-GFP was examined as described in Methods and classified into four patterns: vacuolar membrane (VM), vacuolar membrane + several dots (VM+D), vacuolar lumen + several dots (VL+D), and cytoplasmic distribution + several dots (C+D). The error bars represent the standard deviation of three biological replicates.

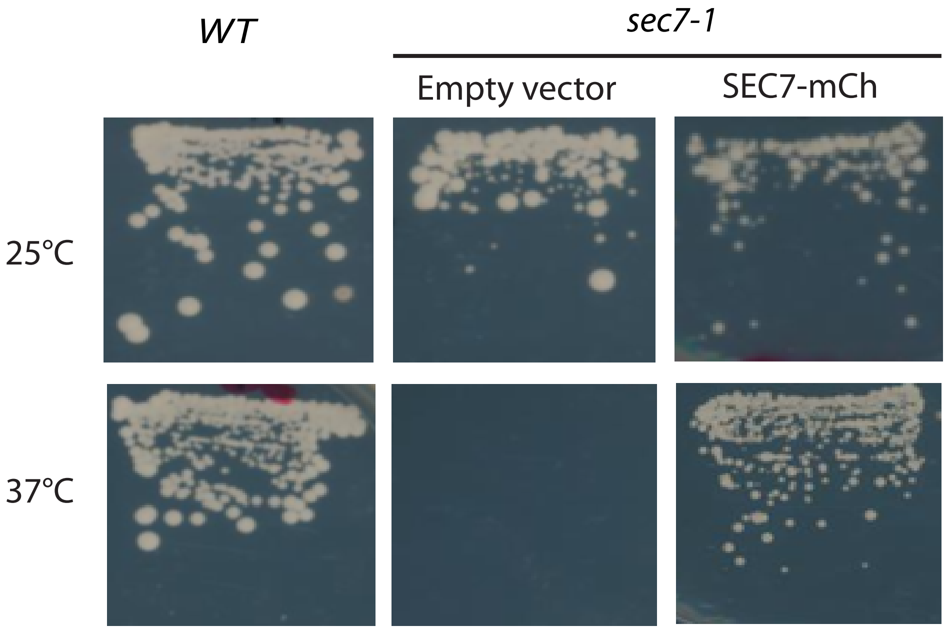


**Figure S8. The Sec7-mCherry construct is functional.** Wild-type strain 23344C (*ura3*) and thermosensitive strain SEY5076 (*MAT sec7-1 ura3-52 leu2-3 leu2-112 SUC2)* transformed with the pBOA010 (*SEC7-mCherry LEU2*) or empty pFL36 (*LEU2*) plasmids were streaked on a glucose-ammonium medium plate containing uracil. The cells were grown for 2 days at 25°C or 37°C.
